# Global research landscape and emerging trends of respiratory microbiota and asthma: a bibliometric analysis

**DOI:** 10.3389/fcimb.2026.1801866

**Published:** 2026-08-06

**Authors:** Qixin Wang, Yongsheng Yu, Baiwu Liang, Lijun Chen, Hongxia Yu, Xianhui Luo, Xiaochao Du, Mo Liao, Haizong Hu, Mei Yang

**Affiliations:** 1 Department of Respiratory and Critical Care Medicine, Dazhou Integrated Traditional Chinese Medicine &Western Medicine Hospital, Dazhou Second People’s Hospital, Dazhou, China; 2 Rehabilitation Center, Dazhou Integrated Traditional Chinese Medicine &Western Medicine Hospital, Dazhou Second People’s Hospital, Dazhou, China; 3 Department of Oncology, Dazhou Integrated Traditional Chinese Medicine &Western Medicine Hospital, Dazhou Second People’s Hospital, Dazhou, China

**Keywords:** asthma, bibliometric analysis, dysbiosis, immune regulation, respiratory microbiota

## Abstract

**Background:**

Asthma is a heterogeneous chronic airway disease in which respiratory microbiota is increasingly recognized as key contributors to airway inflammation, immune regulation, and clinical phenotypes. However, the rapid growth of publications has resulted in a fragmented knowledge landscape, hindering the identification of core contributors and emerging research directions.

**Methods:**

A bibliometric analysis was conducted using publications from the Web of Science Core Collection (WoSCC) and Scopus (2000–2025). Bibliometrix, VOSviewer, and CiteSpace were applied independently to each database to assess publication trends, collaboration networks, co-citation structures, keyword co-occurrence, and burst dynamics, enabling cross-database validation.

**Results:**

A total of 516 WoSCC and 572 Scopus records were included. Both datasets showed consistent growth after 2015, with comparable annual growth rates. The United States, China, and the United Kingdom were leading contributors and central collaboration hubs. Core journals and co-citation patterns were highly concordant across databases. Three reproducible research themes were identified: respiratory microbiota dysbiosis, immune–microbiota interactions, and early-life microbial exposure. Temporal analyses consistently showed a shift toward phenotype-oriented and mechanistic research, with increasing focus on disease severity, immune regulation, and biomarker-related topics.

**Conclusion:**

This dual-database bibliometric study provides a validated overview of the evolving research landscape of respiratory microbiota in asthma. The findings suggest a transition toward more integrative and mechanism-oriented research, offering a structured framework to guide future studies and methodological development.

## Background

Asthma is a heterogeneous chronic inflammatory airway disease characterized by variable airflow limitation, airway hyperresponsiveness, and recurrent respiratory symptoms, affecting more than 300 million individuals worldwide (Pavord et al., 2018; Global Initiative for Asthma, 2025). The disease imposes a substantial socioeconomic burden, with global prevalence rates of 4.3% in adults and a reported 495,100 asthma-related deaths in 2017 (Asher et al., 2020; GBD 2017 Causes of Death Collaborators, 2018; Reddel et al., 2022). Clinically, asthma manifests as recurrent coughing (especially at night or early morning), wheezing, shortness of breath, and chest tightness, usually accompanied by reversible airflow limitation (Global Initiative for Asthma, 2025). Although inhaled corticosteroids and biologic therapies have improved asthma management, a considerable proportion of patients continue to experience poor disease control and frequent exacerbations (Fahy, 2015; Hekking et al., 2015).

In recent years, the respiratory microbiota has emerged as an important factor in airway immune regulation and asthma pathogenesis (Hilty et al., 2010) (Miller et al., 2021; Kozik et al., 2023). Culture-independent techniques, including 16S rRNA gene sequencing and shotgun metagenomics, have revealed distinct microbial communities in healthy individuals and patients with asthma (Charlson et al., 2011; Ver Heul et al., 2019). Accumulating evidence indicates that airway microbial dysbiosis is associated with asthma severity, inflammatory endotypes, and treatment responsiveness (Huang et al., 2022; Li et al., 2023; Li et al., 2024). Several studies have reported enrichment of potentially pathogenic genera, including *Haemophilus, Moraxella, and Streptococcus*, in patients with severe asthma or neutrophilic inflammation, suggesting a link between microbial composition and disease phenotype (Green et al., 2014; Li et al., 2017; Shteinberg et al., 2024). Beyond local airway effects, the concept of the gut–lung axis has emerged, emphasizing potential bidirectional interactions between gut microbiota and respiratory immunity in asthma (Hufnagl et al., 2020; Alharris et al., 2022; Song et al., 2024). Early-life microbial exposures, such as delivery mode, antibiotic use, and early respiratory infections, have been shown to influence immune development and asthma susceptibility (Stern et al., 2020; Miller et al., 2021).

Recent multi-omics studies integrating microbiota, transcriptomic, metabolomic, and immunological data have further revealed complex host–microbe interactions underlying asthma endotypes and disease progression (Logotheti et al., 2021; Kermani et al., 2024; Zhang et al., 2024). However, findings across individual studies remain heterogeneous due to variations in study populations, sampling sites, sequencing platforms, and analytical approaches (Saito et al., 2024; Huang, 2025). Importantly, the expanding volume and heterogeneity of the literature have led to a fragmented and rapidly evolving knowledge structure, making it difficult to systematically evaluate how research themes, methodological approaches, and conceptual focuses have changed over time.

Bibliometric analysis offers a robust quantitative approach to map knowledge structures, collaboration networks, and thematic evolution within a research domain. Therefore, the present study aims to comprehensively characterize the global research landscape of respiratory microbiota in asthma through an integrated bibliometric analysis based on multiple databases.

## Materials and methods

### Data source and study design

This bibliometric study was conducted to systematically analyze global research trends and patterns related to respiratory microbiota in asthma. Relevant literature was retrieved from two major academic databases: the Web of Science Core Collection (WoSCC) and Scopus. The analysis covered publications from 2000 to 2025, and all records were collected on January 11, 2026, to minimize potential bias caused by database updates. Only peer-reviewed original research articles and review articles published in English were included in this study. This study is designed as a descriptive bibliometric mapping analysis rather than a systematic review or meta-analysis, focusing on knowledge structure, research dynamics, and thematic evolution rather than clinical or biological effect estimation.

### Search strategy

To identify relevant publications, a comprehensive search strategy was developed by combining terms related to asthma and respiratory microbiota. Asthma-related terms included “asthma”. Respiratory microbiota-related terms included “respiratory microbiota”, “airway microbiota”, “lung microbiome”, “microbiome”, “microbial community”, “dysbiosis”, and related variants associated with airway microbial ecology. To maintain retrieval specificity, these microbiota-related terms were combined with asthma-related terms using Boolean operators. The search strategy was designed to balance sensitivity and specificity to ensure comprehensive yet accurate retrieval of relevant studies. Detailed and complete search strategies for each database are provided in the Supplementary Materials.

### Data extraction and preprocessing

After retrieving the records, all relevant bibliographic details were exported in formats compatible with bibliometric software. For WoSCC, data were exported in plain text format, while Scopus data were retrieved in CSV format containing author, institution, and keyword information. Before analysis, data cleaning steps were performed, including author name disambiguation, institution standardization, and keyword harmonization. All preprocessing steps followed standardized bibliometric data cleaning procedures to ensure consistency across the two databases. For example, terms such as “respiratory microbiota” and “lung microbiota” were treated as synonymous and combined. Two researchers independently conducted the data cleaning, with discrepancies being resolved through discussion.

### Bibliometric analysis using Bibliometrix

Descriptive statistics were generated using the Bibliometrix R package (version 4.5.1). Data from both WoSCC and Scopus were processed using the convert2df function. Key indicators including publication trends, document types, journals, authorship, country contributions, and citation metrics were analyzed using the biblioAnalysis function. Visualization was performed using built-in plotting functions.

### Network analysis using VOSviewer

Network analysis was performed using VOSviewer to construct co-authorship, co-occurrence, and co-citation networks based on WoSCC and Scopus data. Full counting method was applied consistently across all analyses to ensure comparability between datasets. The co-authorship networks were constructed for authors and countries, and keyword co-occurrence networks were also generated to visualize research topics. For citation network analysis, both co-citation and bibliographic coupling were considered to understand relationships among sources and references. The analysis was conducted using the full counting method, with specific thresholds set to ensure meaningful results. For the WoSCC dataset, minimum publication thresholds in co-authorship analysis were set to ≥4 for countries, ≥4 for institutions, and ≥4 for authors. In WoSCC-based co-citation analysis, the minimum citation threshold for source references was defined as ≥40. For keyword co-occurrence analysis in WoSCC, keywords with a minimum occurrence frequency of ≥7 were included. For the Scopus dataset, the minimum publication thresholds in co-authorship analysis were set to ≥3 for countries, ≥3 for institutions, and ≥4 for authors. In co-citation analysis based on Scopus, the minimum citation count for source references was set to ≥10. For keyword co-occurrence analysis in Scopus, only keywords with an occurrence frequency of ≥30 were included. Different thresholds were selected for WoSCC and Scopus because of differences in database size, indexing structure, and citation density, aiming to optimize network interpretability while preserving representative nodes.

### Burst detection using CiteSpace

Burst detection analysis was performed using CiteSpace to identify keywords or references showing a rapid increase in citation frequency or occurrence intensity during a specific time period. This approach helps detect emerging research frontiers, rapidly evolving topics, and shifts in scientific attention within asthma-related respiratory microbiota research.

### Cross-database validation strategy

To enhance robustness and reproducibility, a cross-database validation strategy was implemented. WoSCC and Scopus were treated as independent data sources. All analyses were conducted separately using identical search strategies, inclusion criteria, and analytical workflows. The decision not to merge WoSCC and Scopus datasets is based on systematic differences in journal coverage, indexing rules, and metadata structures. Direct merging may introduce duplication bias and thematic distortion, potentially affecting network and trend analyses. Therefore, this study adopts an independent analysis followed by cross-validation strategy to improve reproducibility and reliability.

Results were compared to assess consistency in publication trends, collaboration networks, co-citation structures, keyword clustering, and thematic evolution. Only convergent patterns across both databases were emphasized in interpretation. This dual-database design reduces the risk of database-specific indexing bias and enhances the generalizability of bibliometric findings.

This study focuses on mapping research activity and knowledge structures rather than evaluating biological or clinical evidence. Accordingly, all findings should be interpreted as representations of knowledge evolution rather than direct evidence of disease mechanisms.

## Results

### Overview of publication characteristics and data sources

After deduplication, 516 publications from the Web of Science Core Collection (WoSCC) and 572 publications from Scopus met the inclusion criteria and were incorporated into the final bibliometric analysis. The WoSCC dataset spanned 227 journals, whereas the Scopus dataset covered 262 sources, indicating broad and multidisciplinary dissemination of research on respiratory microbiota in asthma. Both databases demonstrated a consistent and rapid increase in annual publication output, with average annual growth rates of 32.35% in WoSCC and 25.24% in Scopus, indicating the accelerating expansion of this research field. The mean document age was 4.38 years in WoSCC and 5.35 years in Scopus, while the average citations per document reached 45.55 and 52.71, respectively (**Supplementary Table 1**), reflecting the relatively recent yet highly influential nature of the literature. In terms of document types, original research articles predominated in both datasets (WoSCC: n = 295; Scopus: n = 312), followed by review articles (WoSCC: n = 213; Scopus: n = 260), demonstrating a balanced structure of primary research and knowledge synthesis. A total of 2,837 authors contributed to WoSCC-indexed publications and 3,003 authors to Scopus-indexed publications. The mean number of authors per document was comparable between the two databases (7.7 in WoSCC and 7.68 in Scopus), highlighting the collaborative nature of this research domain. Internationally co-authored publications accounted for 29.07% of WoSCC records and 25.57% of Scopus records, indicating substantial cross-national collaboration (**Supplementary Table 1**).

Overall, the high degree of consistency between WoSCC and Scopus across publication growth, document types, citation impact, and collaboration patterns supports the robustness and reliability of the dual-database bibliometric framework used in this study.

### Temporal trends in publication output

**Figure 1:**
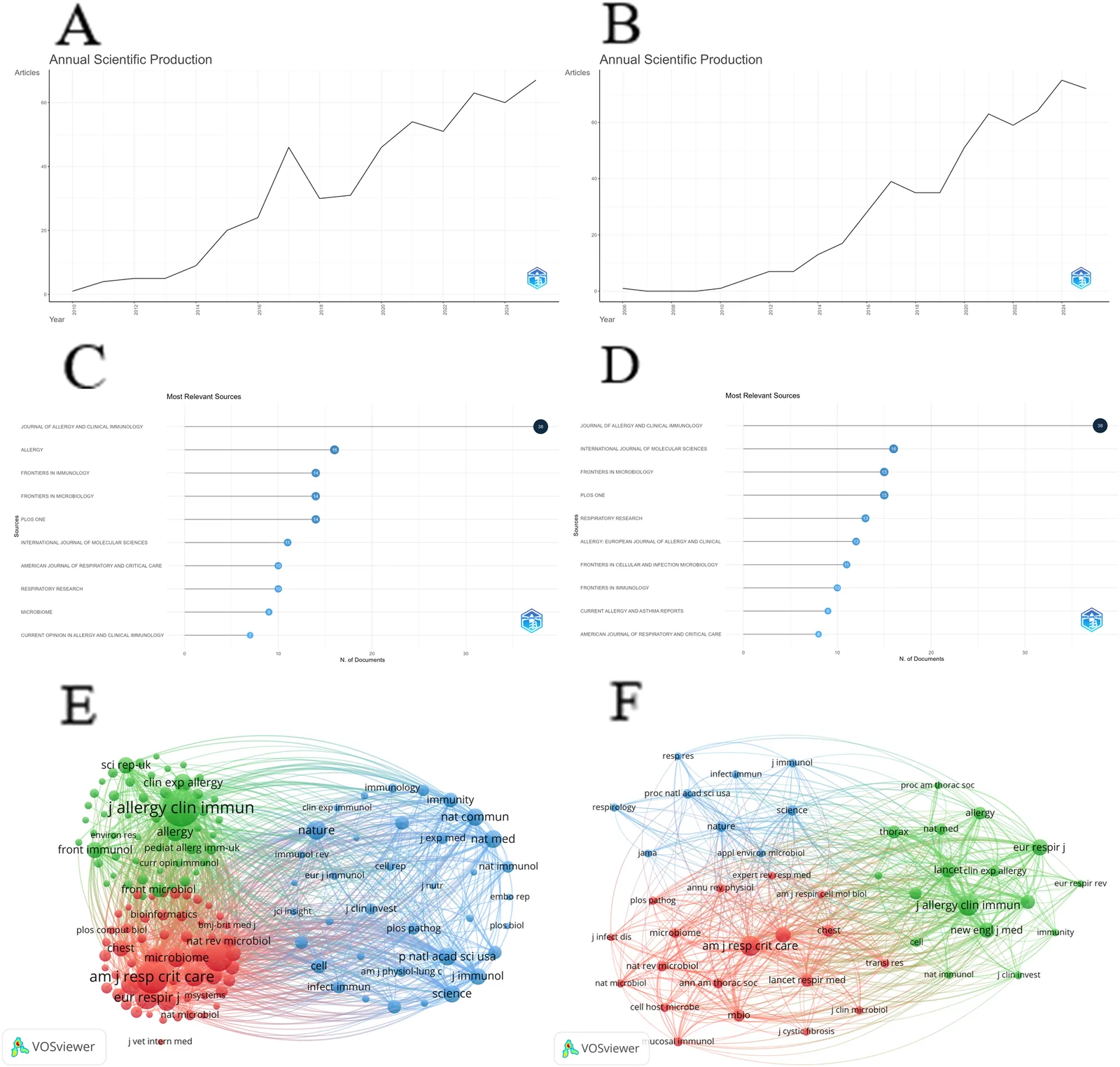
Temporal trends and journal distribution of publications. **(A)** Annual number of publications on respiratory microbiota and asthma indexed in the Web of Science Core Collection (WoSCC). **(B)** Annual number of publications in the Scopus database. **(C)** Most productive journals in the WoSCC dataset based on publication counts. **(D)** Most productive journals in the Scopus dataset. **(E, F)** Journal co-citation networks in WoSCC **(E)** and Scopus **(F)**.

Temporal analysis of publication output revealed a consistent and staged growth pattern in respiratory microbiota research related to asthma across both databases. In the WoSCC dataset, publications were sparse before 2014, increasing from 1 article in 2010 to 9 articles in 2014 (**Figure 1A**; **Supplementary Table 2**), representing an early exploratory phase of the field. A rapid expansion phase followed between 2015 and 2017, during which annual publications rose sharply from 20 to 46 articles. After 2018, research activity entered a sustained high-output period, with annual publications fluctuating between 30 and 67 articles through 2025, peaking in 2025. A comparable trajectory was observed in the Scopus dataset, although with slightly earlier coverage. Sporadic publications appeared before 2010, followed by a marked acceleration after 2015 (**Figure 1B**; **Supplementary Table 2**). Annual output increased from 17 publications in 2015 to 39 in 2017, reaching 51 by 2020. From 2021 onward, publication numbers remained consistently high, exceeding 60 articles per year, with a peak of 75 articles in 2024 and 72 in 2025. Notably, both databases independently suggest three distinct developmental stages: an initial low-output phase, a rapid growth period beginning around 2015, and a sustained high-productivity stage after 2018. The convergence of these patterns across two independent data sources underscores the robustness of the observed expansion and highlights the growing global research interest in the role of respiratory microbiota in asthma.

### Most relevant and co-cited journals

Journal analysis across WoSCC and Scopus consistently identified the Journal of Allergy and Clinical Immunology as the most productive and influential source in respiratory microbiota and asthma research, ranking first in publication output in both databases. Other highly productive journals included Allergy, Frontiers in Microbiology, Frontiers in Immunology, PLoS One, International Journal of Molecular Sciences, and the American Journal of Respiratory and Critical Care Medicine, reflecting broad dissemination across respiratory medicine, allergy–immunology, and microbiota-focused journals (**Figures 1C, D**, **Supplementary Table 3**). Co-citation analysis further showed a stable core knowledge base dominated by leading clinical and translational journals. Sources such as Journal of Allergy and Clinical Immunology, American Journal of Respiratory and Critical Care Medicine, European Respiratory Journal, Thorax, and Nature ranked among the most frequently co-cited journals (**Figures 1E, F**, **Supplementary Table 4**). Notably, substantial overlap was observed between the most productive and most co-cited journals, indicating that publication output in this field is closely aligned with high-impact, clinically relevant research. Overall, the convergence of productivity and co-citation patterns across both databases highlights the interdisciplinary nature of this research area, positioned at the intersection of clinical respiratory medicine, allergy and immunology, and microbiota science.

### Author productivity, impact, and collaboration patterns

**Figure 2:**
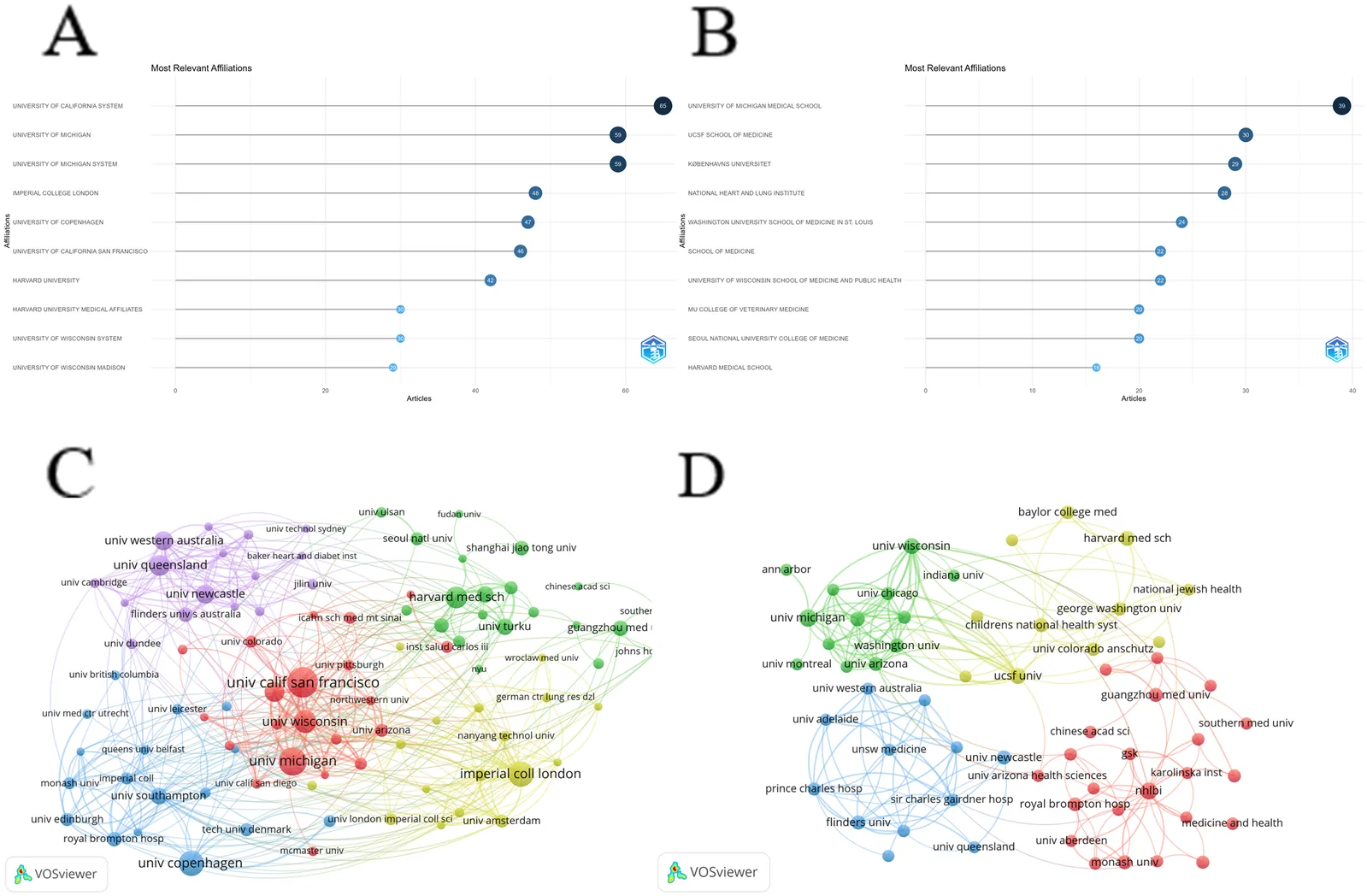
Author productivity, citation impact, and collaboration networks. **(A, B)** Most relevant authors in the WoSCC **(A)** and Scopus **(B)** datasets. **(C, D)** Author collaboration networks constructed using VOSviewer for WoSCC **(C)** and Scopus **(D)**.

Across both WoSCC and Scopus, a highly consistent core author group was identified, with Huang YJ ranking as the most prolific contributor and also showing the highest local citation impact within the WoSCC dataset (**Figures 2A, B**, **Supplementary Table 5**). Other leading authors, including Bisgaard H, Lynch SV, and several consistently productive researchers such as Chung KF, Hasegawa K, and Stokholm J, formed a stable group of high-output contributors, indicating long-term engagement in respiratory microbiota and asthma research. Local citation analysis revealed a strong alignment between publication productivity and scholarly influence. Authors with high publication counts frequently also demonstrated substantial local citation impact, suggesting that leadership in this field is concentrated among a limited number of investigators whose work has shaped the core knowledge structure. Collaboration network analysis further illustrated the presence of well-defined and interconnected research teams (**Figures 2C, D**). In the WoSCC dataset, authors meeting the inclusion threshold formed several major clusters, with the largest centered on Huang YJ, Boushey HA, Lynch SV, and Huffnagle GB, characterized by high total link strength values and dense internal collaboration. Another prominent cluster focused primarily on pediatric asthma and early-life respiratory microbiota, indicating thematic specialization within the broader field. A comparable but more compact collaboration structure was observed in the Scopus dataset, again dominated by central figures such as Huffnagle GB, Dickson RP, Lynch SV, and Huang YJ. Overall, the integration of productivity metrics, local citation impact, and collaboration patterns indicates that research on asthma and respiratory microbiota is driven by a relatively small number of highly influential authors embedded within stable, topic-focused collaborative networks.

### Institutional productivity and collaboration networks

**Figure 3:**
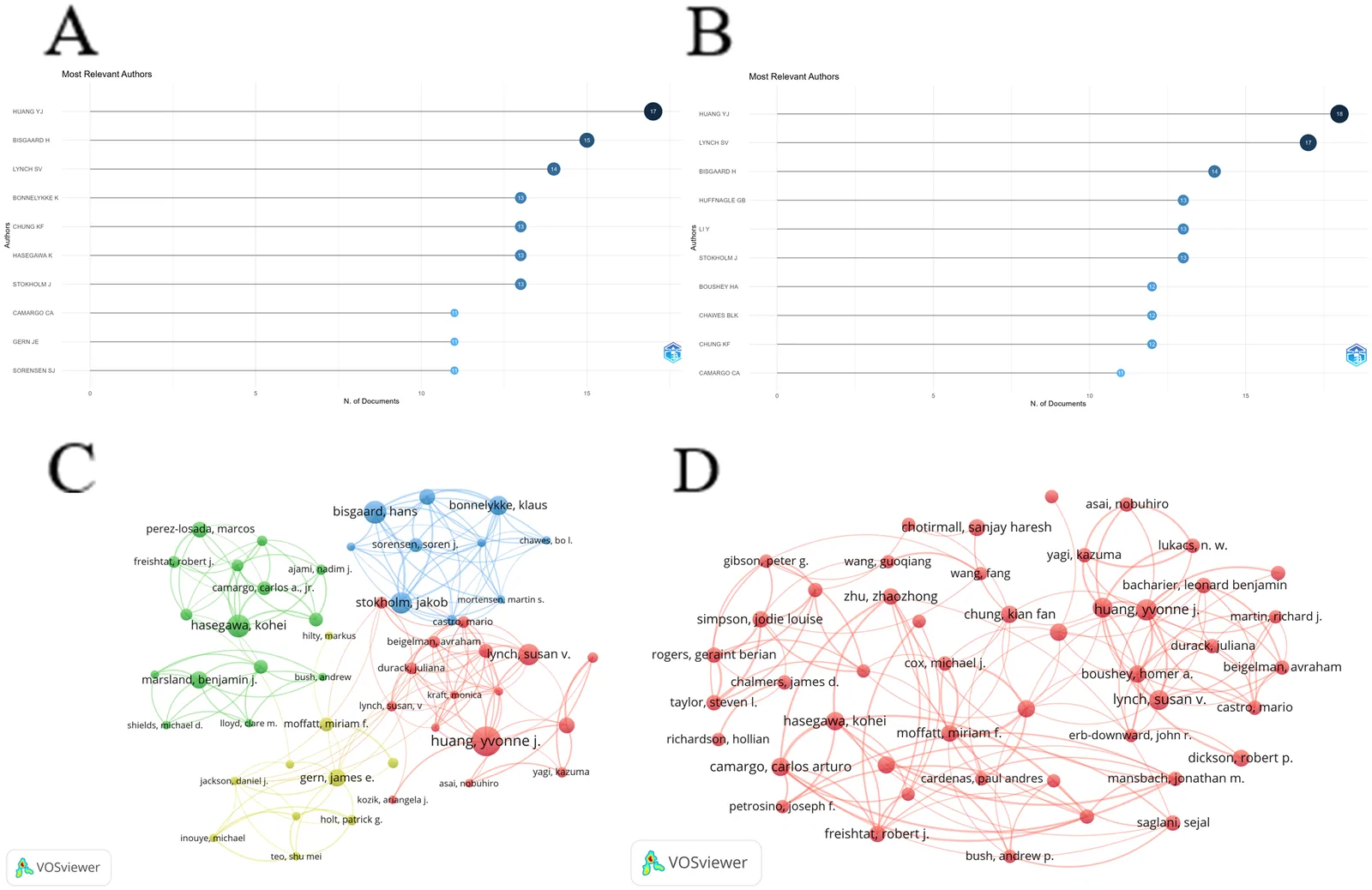
Institutional productivity and collaboration patterns. **(A)** Most relevant institutions in the WoSCC dataset. **(B)** Most relevant institutions in the Scopus dataset. **(C, D)** Institutional collaboration networks derived from WoSCC **(C)** and Scopus **(D)** data using VOSviewer.

Institutional analysis revealed a clearly stratified global research structure dominated by a small group of high-output academic medical centers (**Figures 3A, B**, **Table 1**). In the WoSCC dataset, the University of California system and the University of Michigan (including its affiliated system) emerged as the most productive institutional entities, followed by leading European centers such as Imperial College London and the University of Copenhagen. Several major U.S. academic hubs, including UCSF and Harvard-affiliated institutions, also showed sustained high research output. Beyond North America and Western Europe, institutions from Asia and Australia—including Seoul National University, the University of Turku, and the University of Queensland—showed growing participation, indicating an increasingly diversified global research landscape. A similar institutional hierarchy was observed in the Scopus dataset, where major U.S. and European clinical–academic centers again occupied central positions. Despite differences in institutional naming granularity between databases, the core group of leading institutions remained highly consistent, underscoring the robustness of the identified research hubs across data sources. Collaboration network analysis further demonstrated that institutional contributions were not evenly distributed but instead organized into several densely connected clusters (**Figures 3C, D**). In the WoSCC network, five major institutional clusters were identified, each anchored by high–link-strength hubs such as Imperial College London and UCSF, suggesting the presence of stable, long-term collaborative consortia. The Scopus-based network displayed a comparable modular structure, with institutions like the University of Michigan and National Jewish Health acting as influential nodes with strong citation impact, reflecting both productivity and scientific influence.

**Table 1:** Top 10 most relevant affiliations based on WoSCC and Scopus database.

WoSCC		Scopus	
Affiliation	Articles	Affiliation	Articles
University of California System	65	University of Michigan Medical School	39
University of Michigan	59	UCSF School of Medicine	30
University of Michigan System	59	KØbenhavns Universitet	29
Imperial College London	48	National Heart and Lung Institute	28
University of Copenhagen	47	Washington University School of Medicine in ST. Louis	24
University of California San Francisco	46	University of Wisconsin School of Medicine and Public Health	22
Harvard University	42	MU College of Veterinary Medicine	20
Harvard University Medical Affiliates	30	Seoul National University College of Medicine	20
University of Wisconsin System	30	Harvard Medical School	16
University of Wisconsin Madison	29	National Taiwan University Hospital	16

Overall, these findings indicate that global research on asthma and respiratory microbiota is shaped by a relatively small number of highly interconnected core institutions that function as collaborative and intellectual hubs. At the same time, a growing number of emerging institutions from Asia and other regions are progressively integrating into these established networks, pointing to a gradual broadening of the global research ecosystem.

### Country productivity, citation impact, and international collaboration networks

**Figure 4:**
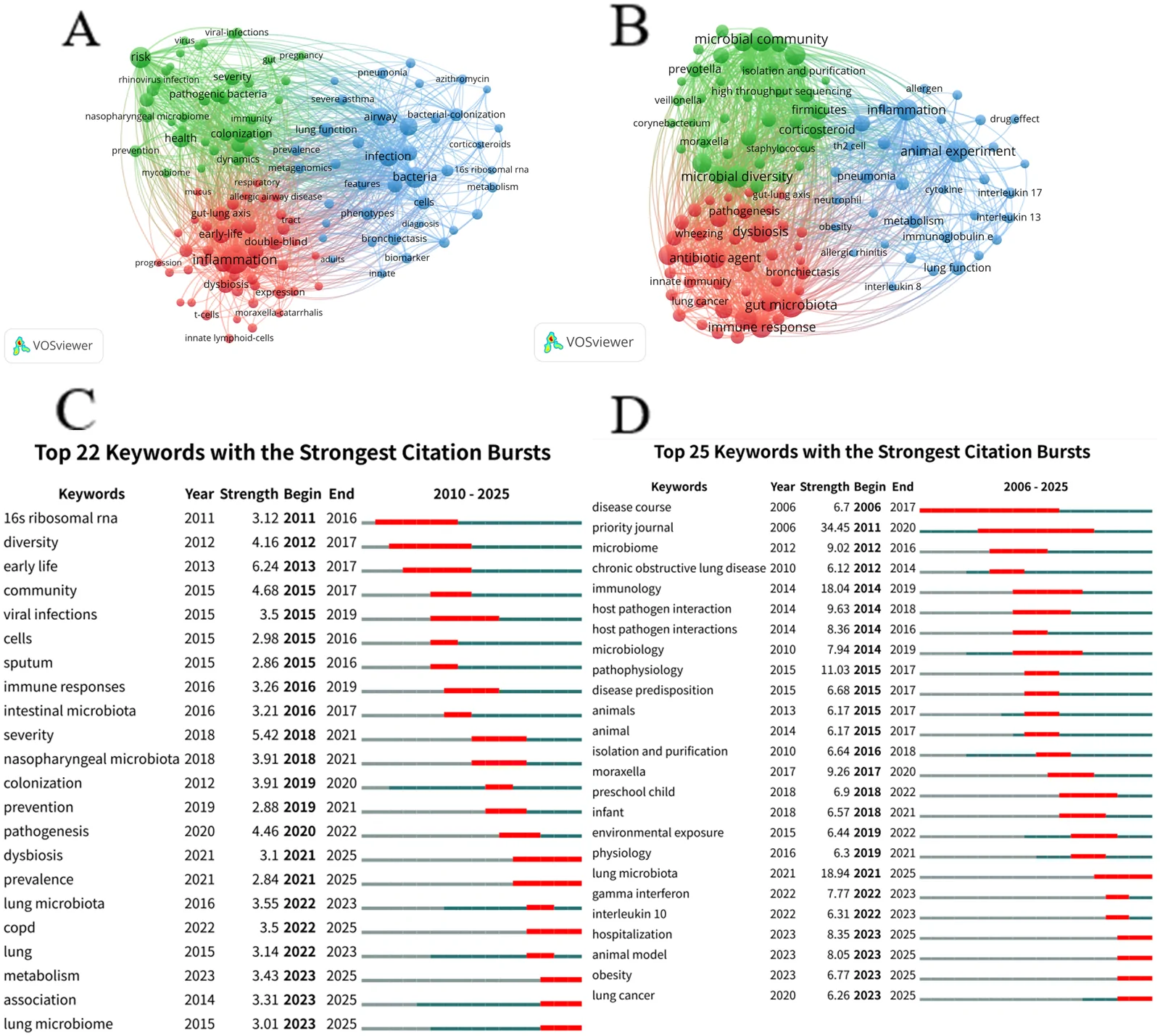
Country productivity, citation impact, and international collaboration. **(A, B)** Country-level publication output and citation impact based on corresponding author analysis in WoSCC **(A)** and Scopus **(B)**. **(C, D)** International collaboration networks of countries generated from WoSCC **(C)** and Scopus **(D)** datasets.

Corresponding author analysis highlighted a concentrated global research landscape in respiratory microbiota and asthma (**Figures 4A, B**, **Supplementary Table 6**). The United States led both publication output (WoSCC: 136 articles, 26.4%; Scopus: 122 articles, 21.4%) and total citations (WoSCC: 6,937; Scopus: 8,849), establishing itself as the dominant scientific hub. China followed closely in publication volume, while several European countries—including the United Kingdom, Denmark, Finland, and Switzerland—showed high citation impact despite relatively lower output, indicating that these nations contribute disproportionately influential research. Analysis of multiple-country publications (MCP) revealed that the United Kingdom (WoSCC: 57.1%; Scopus: 35.0%) and the Netherlands (Scopus: 77.8%) are highly collaborative, reflecting strong engagement in international research networks. VOSviewer-based network mapping further illustrated the structure of global collaborations (**Figures 4C, D**). In WoSCC, 30 countries formed two major clusters: the first, led by the United States, United Kingdom, Australia, Germany, and France, exhibited dense interconnections with high total link strength values (e.g., England TLS = 119; USA TLS = 114), indicating stable and sustained collaborations. The second cluster, encompassing China, Japan, South Korea, Spain, and Southern European countries, demonstrated geographically influenced collaboration patterns. Scopus networks were denser, with the United States (TLS = 101) and United Kingdom (TLS = 103) acting as central hubs connecting multiple countries across Europe, Asia, and Oceania. Collectively, these analyses indicate that global research on respiratory microbiota in asthma is concentrated in a few high-output, highly interconnected countries, while international collaborations facilitate the dissemination of influential findings across regions.

### High-impact literature landscape

**Figure 5:**
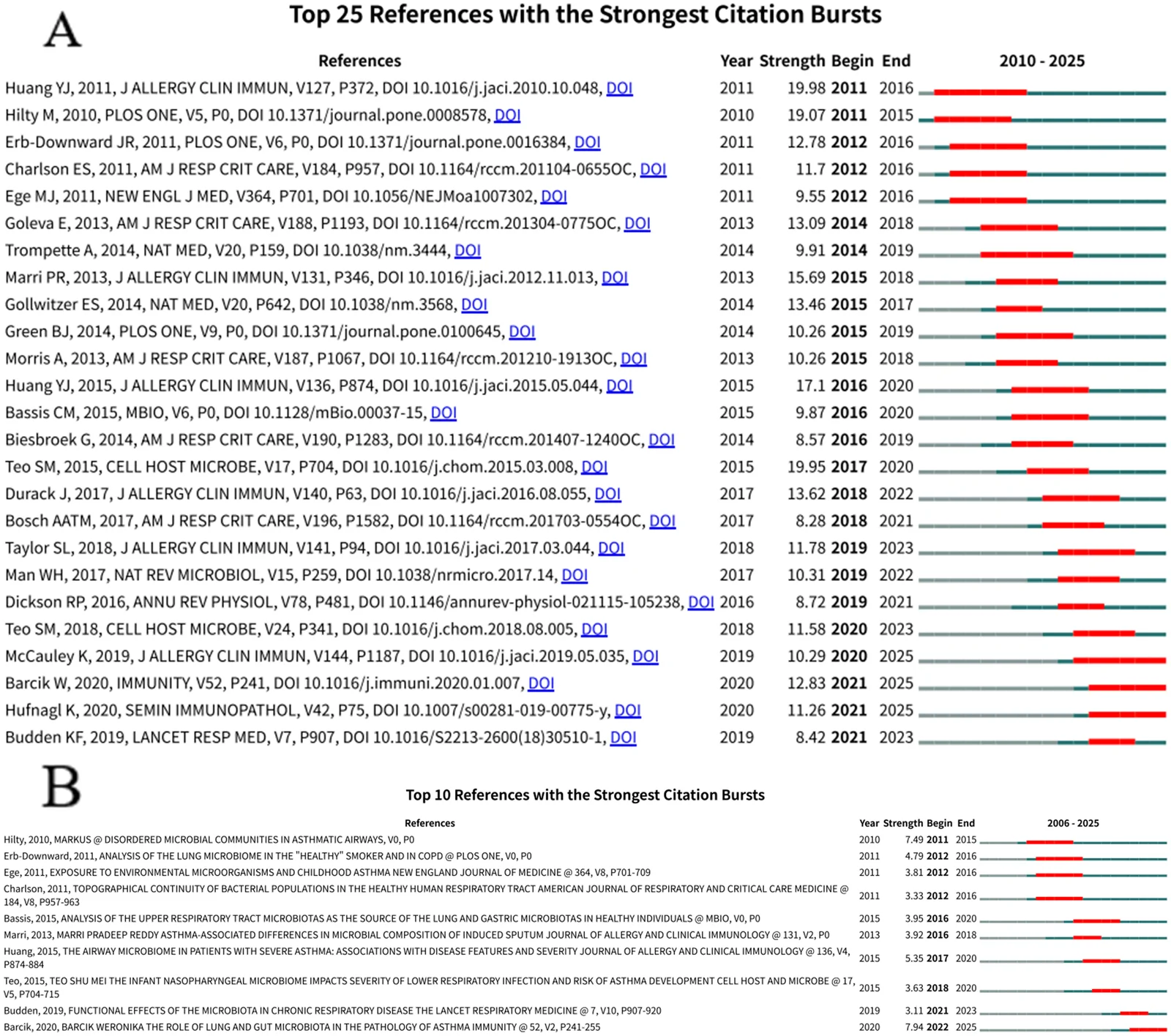
High-impact literature and citation burst analysis. Visualization of highly cited references and citation burst patterns in asthma-related respiratory microbiota research in WoSCC **(A)** and Scopus **(B)**.

Analysis of highly cited publications revealed a clear convergence between foundational studies and temporally concentrated citation bursts, reflecting both enduring academic influence and evolving research priorities in respiratory microbiota and asthma (**Table 2**, **Figure 5**). In both WoSCC and Scopus datasets, Hilty et al. (2010) was the most influential work, with 1,363 citations in WoSCC (80.18 citations/year) and 1,515 citations in Scopus (89.12 citations/year), showing strong citation bursts during 2011–2015 and demonstrating both immediate and sustained impact. Similarly, Huang et al. (2011), which examined respiratory microbiota and bronchial hyperresponsiveness, had high cumulative citations (560 in WoSCC; 624 in Scopus) and the strongest citation burst in WoSCC (strength = 19.98, 2011–2016), highlighting its role in linking microbial composition to asthma pathophysiology. Mechanistic and immunological studies also formed a major component of the high-impact landscape. Larsen (2017) achieved the highest annual citation rate (WoSCC: 102.0; Scopus: 108.8 citations/year), while Gollwitzer et al. (2014) and Barcik et al. (2020) showed sustained citation bursts extending into recent years. Several review and synthesis articles, including Dickson et al. (2015, 2016) and Budden et al. (2019), displayed moderate-to-high total citations alongside late-onset citation bursts after 2019, reflecting a field-wide shift from descriptive microbiota profiling toward integrative and translational frameworks.

**Table 2:** Top 10 cited references related to the relationship between asthma and respiratory microbiota.

WoSCC			
Paper	Title	Total citations	TC per Year
HILTY M, 2010, PLOS ONE	Disordered microbial communities in asthmatic airways	1363	80.18
LARSEN JM, 2017, IMMUNOLOGY	The immune response to Prevotella bacteria in chronic inflammatory disease	1020	102.00
HUANG YJ, 2011, J ALLERGY CLIN IMMUN	Respiratory microbiota and bronchial hyperresponsiveness in patients with suboptimally controlled asthma	560	35.00
GOLLWITZER ES, 2014, NAT MED	Lung microbiota promotes tolerance to allergens in neonates via PD-L1	472	36.31
SZE MA, 2012, AM J RESP CRIT CARE	The lung tissue microbiota in chronic obstructive pulmonary disease	423	28.20
HUANG YJ, 2015, J ALLERGY CLIN IMMUN-a	The airway microbiota in patients with severe asthma: Associations with disease features and severity	401	33.42
HUFFNAGLE GB, 2017, MUCOSAL IMMUNOL	The respiratory tract microbiota and lung inflammation: a two-way street	382	38.20
JARTTI T, 2017, J ALLERGY CLIN IMMUN	Role of viral infections in the development and exacerbation of asthma in children	353	35.30
BUDDEN KF, 2019, LANCET RESP MED	Functional effects of the microbiota in chronic respiratory disease	322	40.25
ANAND S, 2018, FRONT MICROBIOL	Diet, Microbiota and Gut-Lung Connection	299	33.22
Scopus			
Paper	Title	Total Citations	TC per Year
HILTY M, 2010, PLOS ONE	Disordered microbial communities in asthmatic airways	1515	89.12
LARSEN JM, 2017, IMMUNOLOGY	The immune response to Prevotella bacteria in chronic inflammatory disease	1088	108.80
DICKSON RP, 2016, ANNU REV PHYSIOL	The Microbiota and the Respiratory Tract	745	67.73
HUANG YJ, 2011, J ALLERGY CLIN IMMUNOL	Respiratory microbiota and bronchial hyperresponsiveness in patients with suboptimally controlled asthma	624	39.00
ENAUD R, 2020, FRONT CELL INFECT MICROBIOL	The Gut-Lung Axis in Health and Respiratory Diseases: A Place for Inter-Organ and Inter-Kingdom Crosstalks	579	82.71
BARCIK W, 2020, IMMUNITY	The Role of Lung and Gut Microbiota in the Pathology of Asthma	499	71.29
SZE MA, 2012, AM J RESPIR CRIT CARE MED	The lung tissue microbiota in chronic obstructive pulmonary disease	476	31.73
STERN JL, 2020, SEMINIMMUNOPATHOL	Asthma epidemiology and risk factors	455	65.00
DICKSON RP, 2015, PLOS PATHOG	The Lung Microbiota: New Principles for Respiratory Bacteriology in Health and Disease	436	36.33
HUANG YJ, 2015, J ALLERGY CLIN IMMUNOL	The airway microbiota in patients with severe asthma: Associations with disease features and severity	427	35.58

Overall, the high-impact literature suggests a coherent evolution: early descriptive studies of airway microbial communities laid the groundwork, followed by mechanistic insights into immune–microbiota interactions, and culminating in recent integrative concepts such as the gut–lung axis and asthma phenotype stratification. This progression highlights the maturation of research from observational to mechanistic and translational inquiry.

### Keyword clustering and burst analysis across databases

**Figure 6:**
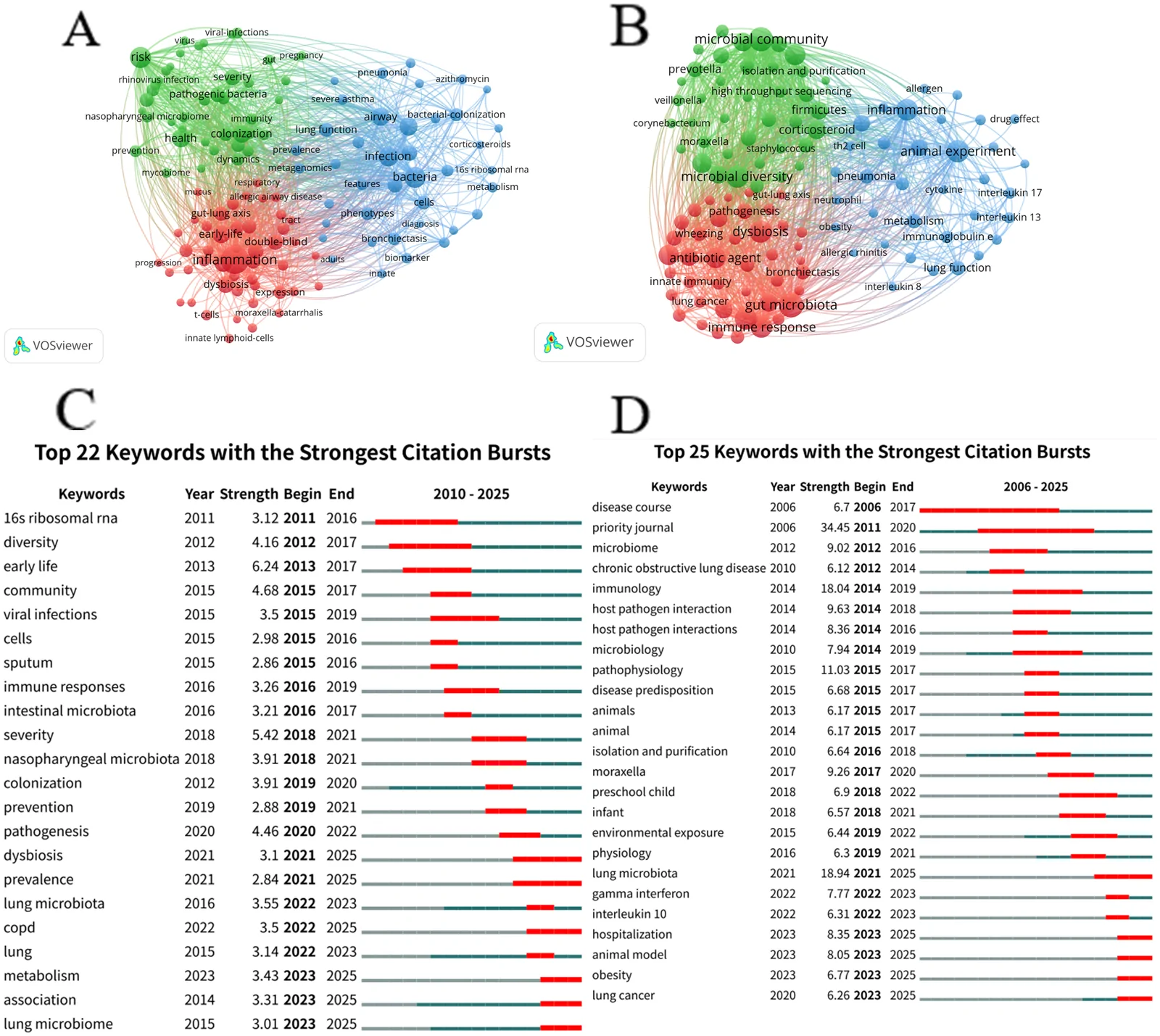
Keyword clustering and burst detection across databases. Keyword co-occurrence clusters generated by VOSviewer in WoSCC **(A)** and Scopus **(B)**and burst detection results in WoSCC **(C)** and Scopus **(D)**.

**Figure 6** presents integrated keyword clustering and burst detection to illustrate the thematic structure and temporal evolution of respiratory microbiota research across WoSCC and Scopus. VOSviewer clustering in both databases revealed a shared core structure focused on microbial composition, host immune responses, and disease-related outcomes. Distinct patterns were observed between the two datasets: in WoSCC, clusters were more strongly disease-oriented, featuring terms such as “COPD,” “lung microbiota,” and “disease severity,” whereas Scopus clusters were enriched with disciplinary and methodological keywords, including “microbiology,” “immunology,” and “host–pathogen interaction,” indicating broader interdisciplinary integration. CiteSpace burst detection highlighted temporal differences. Early bursts in WoSCC were linked to microbial identification and sampling methods, while more recent bursts emphasized “dysbiosis,” “lung microbiota,” and “COPD.” In Scopus, strong early bursts occurred for high-level disciplinary terms such as “microbiota” and “immunology,” with later bursts shifting toward clinical translation and comorbidity-focused topics, including “hospitalization” and “lung cancer.” Notably, burst keywords from both databases aligned with their corresponding VOSviewer clusters, demonstrating spatial–temporal consistency in theme evolution.

Overall, WoSCC and Scopus provide complementary insights, with WoSCC emphasizing disease-specific trends and Scopus highlighting methodological and translational development, together capturing the diversification of respiratory microbiota research over time.

### Thematic evolution of research topics over time

**Figure 7:**
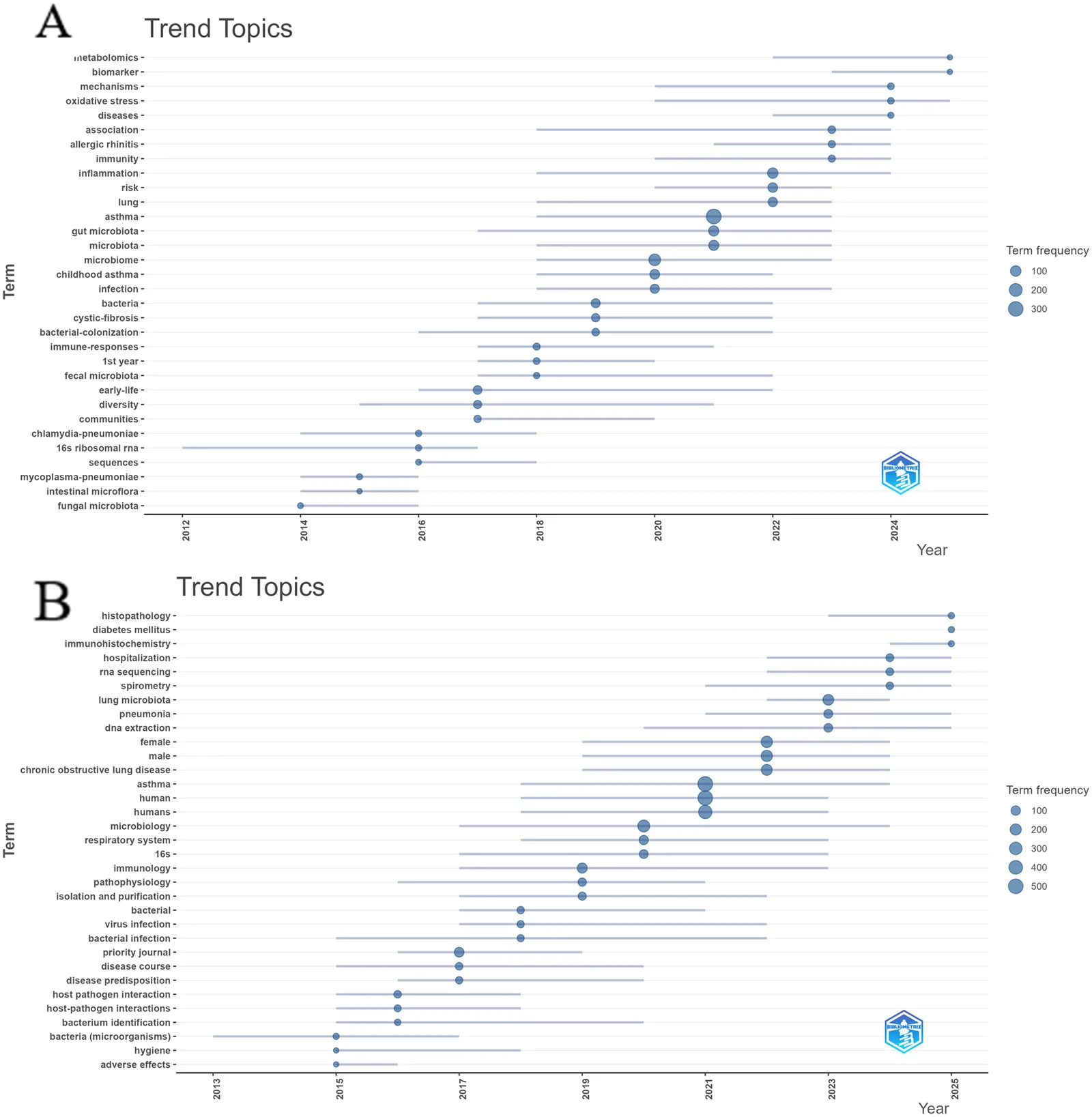
Thematic evolution of research topics over time. Thematic evolution map showing temporal changes in major research topics related to respiratory microbiota and asthma across different periods in WoSCC **(A)** and Scopus **(B)**. Themes are positioned according to their relative importance and temporal emergence in WoSCC and Scopus datasets. Node size represents publication or citation frequency, whereas line thickness and color indicate collaboration strength or clustering relationships.

Trend topic analysis (**Figure 7**) revealed a clear temporal evolution in respiratory microbiota research, progressing from methodological exploration to disease-oriented and translational themes. In the early stage (2012–2016), WoSCC publications were dominated by methodological and pathogen-focused topics, including 16S ribosomal RNA (median year 2016), fungal microbiota (2014), and specific pathogens such as Mycoplasma pneumoniae and Chlamydia pneumoniae (2015–2016). During 2017–2020, WoSCC research shifted toward early-life exposures and community-level microbiota characteristics, with high-frequency terms such as early-life (45), diversity (33), communities (18), and immune responses (15), reflecting growing interest in host–microbiota interactions. In contrast, Scopus during this intermediate period emphasized broader biological and clinical contexts, including host–pathogen interaction (32), immunology (133), pathophysiology (55), and microbiology (254), indicating stronger integration with general biomedical research. In the most recent period (2021–2025), both databases converged on disease- and translation-oriented topics. WoSCC highlighted asthma outcomes and mechanisms, with high-frequency terms such as asthma (311, median year 2021), childhood asthma (72), inflammation (94), and emerging terms like biomarker and metabolomics (Q3 = 2025). Scopus, meanwhile, emphasized clinical phenotypes and disease burden, including chronic obstructive lung disease (183), lung microbiota (176, median year 2023), hospitalization (40), and clinical assessment terms such as spirometry and histopathology, underscoring a clinically oriented focus. Together, these findings illustrate a systematic shift from descriptive and methodological studies toward mechanistic, phenotype-driven, and translational research across the field. The proportion of phenotype-related and mechanistic terms increased in recent years, indicating a progressive shift in research focus.

### Integrated hotspot analysis

The integrated hotspot analysis identified three major research directions in respiratory microbiota studies in asthma. First, microbiota composition and diversity emerged as a central focus, supported by frequent terms such as microbiota, microbiota, bacteria, 16S rRNA, diversity, and bacterial colonization across both WoSCC and Scopus, with VOSviewer clusters showing strong co-occurrence. Second, host immunity and inflammatory responses were consistently highlighted, indicated by keywords including immune responses, inflammation, immunology, and pathogenesis, showing significant burst strengths in CiteSpace and increasing frequency over time. Third, early-life exposures and disease phenotypes formed a distinct theme, evidenced by co-occurring terms such as early-life, childhood asthma, risk, association, and prevalence in both databases, reflecting growing attention to the impact of early-life microbiota on disease development. Together, these findings suggest that current research primarily focuses on the dynamic interplay between microbial communities, host immune mechanisms, and early-life determinants, highlighting key areas for future mechanistic and translational studies.

## Discussion

This study was designed to examine whether respiratory microbiota research in asthma has undergone a conceptual transition from descriptive microbial profiling toward phenotype-oriented and mechanistic investigations. By integrating two independent bibliometric datasets, our findings provide converging evidence that the field has progressively shifted in this direction. Early studies were primarily characterized by taxonomic identification and diversity assessment, whereas recent research increasingly emphasizes host–microbiota interactions, immune regulation, and phenotype-associated microbial patterns, supporting a measurable shift in research focus. These results support our initial analytical premise and provide a structured interpretation of the evolving research landscape.

### Methodological strengths and cross-database robustness

Compared with previous bibliometric studies (Sun et al., 2023; Cao et al., 2024; Peng et al., 2024) that mainly relied on single-database analyses, the present study implemented an independent cross-database validation strategy using both WoSCC and Scopus. This approach improves robustness and reduces the likelihood that major trends are driven by database-specific indexing bias or journal coverage differences. The high degree of convergence observed across two independent datasets supports the reproducibility of the identified thematic structures and temporal evolution patterns. Unlike systematic reviews or meta-analyses, bibliometric approaches focus on knowledge structure and research dynamics rather than direct biological or clinical evidence synthesis.

### Dual-database integration and complementary research structures

Unlike previous bibliometric studies relying on a single database (Zhen et al., 2022; Shu et al., 2024), this study provides a more robust evidence framework through cross-validation of two independent databases. By integrating publications from both WoSCC and Scopus, this study enables cross-database validation of research patterns and improves the robustness of trend identification. Despite differences in indexing strategies and disciplinary coverage, both databases demonstrated highly consistent growth trajectories and core thematic structures. By conducting all analyses independently and comparing the resulting knowledge structures, we verified the reproducibility of major research trends and thematic evolution. This convergence reduces the likelihood that findings are driven by database-specific biases and strengthens the methodological rigor of the study.

### Respiratory microbiota dysbiosis as a persistent research hotspot

Respiratory microbiota dysbiosis emerged as a central and persistent research focus across both databases. Consistent reports of reduced microbial diversity and enrichment of Proteobacteria in asthma further support the central role of dysbiosis in disease heterogeneity (Durack et al., 2017; Hufnagl et al., 2020; Diver et al., 2022). Importantly, previous studies emphasize that dysbiosis should be interpreted as a dynamic ecological imbalance rather than a fixed microbial signature, which may explain the heterogeneity observed across studies (Hufnagl et al., 2020; Rick et al., 2020; Baehren et al., 2022). This conceptualization aligns with increasing recognition of microbial contributions to airway inflammation and heterogeneity. The consistency of this theme across datasets suggests that dysbiosis represents a stable and field-defining research direction. Mechanistically, dysbiosis may contribute to persistent airway inflammation by altering local immune microenvironment and microbial metabolite profiles.

Importantly, several dysbiosis-associated patterns identified in this analysis, including Proteobacteria enrichment, Haemophilus predominance, and neutrophilic inflammation, are not unique to asthma and have also been widely reported in COPD and bronchiectasis. Previous comparative and bibliometric studies suggest that these chronic airway diseases may share a broader ecological pattern characterized by reduced microbial diversity, expansion of potentially pathogenic taxa, and altered host–microbe interactions. The overlap of these thematic trends indicates that asthma microbiome research is developing within a wider conceptual framework of chronic airway inflammatory diseases. Nevertheless, asthma research remains distinguished by its stronger emphasis on phenotype stratification, immune heterogeneity, and early-life microbial influences, which represent key evolving directions within the field.

### Microbiota–phenotype coupling and thematic maturation

Another major hotspot revealed by keyword clustering is the close association between respiratory microbiota composition and asthma inflammatory phenotypes. Neutrophilic asthma has been repeatedly linked to dominance of Haemophilus influenzae and other Proteobacteria, which are associated with corticosteroid resistance and persistent airway inflammation (Ackland et al., 2022; Brown et al., 2022; Lea et al., 2023; Versi et al., 2023). In contrast, several studies (Li et al., 2017; Liu et al., 2025) have reported relatively higher microbial diversity in eosinophilic or T2-high asthma phenotypes; however, these findings remain heterogeneous and may be influenced by corticosteroid exposure, disease severity, and sampling methodology. In addition, the contribution of oral commensal taxa such as Prevotella further complicates interpretation of microbiota signatures in T2 inflammation. Trend and burst analyses further indicate an increasing focus on disease severity, immune heterogeneity, and clinically relevant phenotypes. This shift suggests a transition toward more translationally meaningful and hypothesis-driven research.

### Immune–microbiota interactions as a mechanistic frontier

Burst keyword analysis revealed a rapid increase in immune-related terms, including “innate immunity,” “Th17,” and “immune regulation,” in recent years. Experimental evidence indicates that respiratory microbiota can modulate host immune responses through multiple pathways, including microbial metabolites and epithelial signaling (Barcik et al., 2020; Hufnagl et al., 2020; Sommariva et al., 2020). The articles increasingly propose that immune–microbiota interactions provide mechanistic explanations for the coexistence of multiple asthma phenotypes within similar clinical severity categories (Lukacs and Huang, 2020; Wang et al., 2024; Yu et al., 2025). This pattern reflects a broader transition toward mechanistic interpretation beyond association-based observations. Thus, immune–microbiota interactions may serve as a key mechanistic bridge linking microbial ecological shifts to clinical phenotype heterogeneity.

### Early-life respiratory microbiota and disease susceptibility

Early-life respiratory microbiota has emerged as a rapidly expanding research theme. Birth cohort studies indicate that early microbial colonization patterns, particularly following viral infections or antibiotic exposure, are strongly associated with subsequent asthma development (Arrieta et al., 2015; van Meel et al., 2017; Depner et al., 2020). The studies emphasize that this developmental window represents a critical opportunity for preventive microbiota-based interventions (Steininger et al., 2023; Zar et al., 2024; Zhu, 2024). This trend reflects growing attention to developmental windows in microbiota–immune interactions.

### Translational challenges and methodological heterogeneity

Despite increasing interest in microbiota-targeted therapies, clinical translation remains limited and inconsistent. Macrolides may alter airway microbial composition through both direct antimicrobial activity and indirect immunomodulatory effects, whereas probiotics and biologics may influence microbial ecology through host immune regulation (O'Mahony, 2015; Segal et al., 2016; Reddel et al., 2022). Although these interventions may reduce exacerbation frequency in selected patients, concerns remain regarding antimicrobial resistance and long-term ecological alterations within the respiratory microbiome.

### Clinical and research implications

The present findings also have several important clinical and research implications. From a clinical perspective, increasing attention to respiratory microbiota reflects a growing recognition that asthma heterogeneity may be partially linked to distinct host–microbe interaction patterns rather than a simple pathogen-centered model. This perspective may contribute to improved phenotype stratification and future precision medicine strategies. From a research perspective, the identified thematic evolution suggests that future studies should prioritize longitudinal cohort designs, phenotype-stratified analyses, and multi-omics integration to better clarify causal mechanisms linking microbial ecology with immune dysfunction and disease progression.

## Limitations and future directions

Several limitations of this study should be acknowledged. First, although publications from the Web of Science Core Collection and Scopus were integrated to enhance data completeness and robustness, relevant studies indexed in other databases or published in regional and non-English journals may not have been fully captured. Second, bibliometric indicators primarily reflect research activity, collaboration patterns, and knowledge structures rather than direct biological mechanisms or clinical effectiveness. Therefore, the findings should be interpreted as mapping intellectual trends rather than establishing causal inferences. Third, keyword- and citation-based analyses inevitably simplify complex biological concepts, particularly in a heterogeneous disease such as asthma, where microbial signatures and host responses vary substantially across populations and phenotypes. High keyword frequency reflects research attention rather than biological importance, and underrepresentation does not imply limited clinical relevance.

Despite these limitations, bibliometric analysis remains a valuable systems-level approach for characterizing research landscapes and identifying emerging directions. Future studies should integrate bibliometric insights with longitudinal cohort data, multi-omics profiling, and experimental validation to bridge the gap between descriptive knowledge structures and mechanistic or translational understanding. In addition, increased methodological standardization in sampling strategies, sequencing platforms, and analytical pipelines will be essential to improve cross-study comparability and reproducibility. Collectively, these efforts may facilitate the identification of clinically relevant microbial targets and support the integration of microbiota research into precision asthma management.

Taken together, three major and consistent shifts in research focus can be identified across both datasets. First, research focus has evolved from descriptive microbial profiling toward integrative analyses of host–microbiota interactions. Second, there is an increasing emphasis on phenotype-oriented investigations, particularly in relation to disease severity and immune heterogeneity. Third, early-life microbial exposure has emerged as a rapidly growing theme, reflecting a shift toward developmental and preventive perspectives. Importantly, these patterns were consistently observed across both WoSCC and Scopus datasets, supporting the robustness and reproducibility of the identified research trends.

In conclusion, this study demonstrates a clear transition toward phenotype-driven and mechanistic paradigms in respiratory microbiota research in asthma. These findings provide a conceptual framework for future studies and support the integration of microbiota research into precision medicine.

## Data Availability

The data presented in the study are deposited in the Figshare, https://doi.org/10.6084/m9.figshare.33086348. Further inquiries can be directed to the corresponding author.
