# Supplementary material for: Global research landscape and emerging trends of respiratory microbiota and asthma: a bibliometric analysis
Source: Front Cell Infect Microbiol. 2026 Aug 6;16:1801866. doi: 10.3389/fcimb.2026.1801866 (PMC13491430; doi:10.3389/fcimb.2026.1801866)
Supplement: Supplementary file 1 [file DataSheet1.docx]

Search Strategy

The WoSCC search strategy was as follows: TS = (asthma AND ("airway microbiome" OR "lung microbiome" OR "respiratory microbiome" OR "airway microbiota" OR "lung microbiota" OR "respiratory microbiota" OR "airway microorganism" OR "lung microorganism" OR "respiratory microorganism" OR "airway microbe" OR "lung microbe" OR "respiratory microbe" OR "airway germ" OR "lung germ" OR "respiratory germ" OR "airway microbiology" OR "lung microbiology" OR "respiratory microbiology" OR "alveolar microbiome" OR "alveolar microbiota" OR "alveolar microorganism" OR "alveolar microbe" OR "alveolar germ" OR "alveolar microbiology" OR "sputum microbiome" OR "sputum microbiota" OR "sputum microorganism" OR "sputum microbe" OR "sputum germ" OR "sputum microbiology" OR "pulmonary microbiome" OR "pulmonary microbiota" OR "pulmonary microorganism" OR "pulmonary microbe" OR "pulmonary germ" OR "pulmonary microbiology" OR "lung tissue microbiome" OR "lung tissue microbiota" OR "lung tissue microorganism" OR "lung tissue microbe" OR "lung tissue germ" OR "lung tissue microbiology"OR "bronchial microbiome" OR "bronchial microbiota" OR "bronchial microorganism" OR "bronchial microbe" OR "bronchial germ" OR "bronchial microbiology" OR "bronchic microbiome" OR "bronchic microbiota" OR "bronchic microorganism" OR "bronchic microbe" OR "bronchic germ" OR "bronchic microbiology")) AND LA = (English) AND DT = (Article OR Review) AND DOP = (2000-01-01/2025-12-31).

The Scopus search strategy was: TITLE-ABS-KEY (asthma AND ("airway microbiome" OR "lung microbiome" OR "respiratory microbiome" OR "airway microbiota" OR "lung microbiota" OR "respiratory microbiota" OR "airway microorganism" OR "lung microorganism" OR "respiratory microorganism" OR "airway microbe" OR "lung microbe" OR "respiratory microbe" OR "airway germ" OR "lung germ" OR "respiratory germ" OR "airway microbiology" OR "lung microbiology" OR "respiratory microbiology" OR "alveolar microbiome" OR "alveolar microbiota" OR "alveolar microorganism" OR "alveolar microbe" OR "alveolar germ" OR "alveolar microbiology" OR "sputum microbiome" OR "sputum microbiota" OR "sputum microorganism" OR "sputum microbe" OR "sputum germ" OR "sputum microbiology" OR "pulmonary microbiome" OR "pulmonary microbiota" OR "pulmonary microorganism" OR "pulmonary microbe" OR "pulmonary germ" OR "pulmonary microbiology" OR "lung tissue microbiome" OR "lung tissue microbiota" OR "lung tissue microorganism" OR "lung tissue microbe" OR "lung tissue germ" OR "lung tissue microbiology" OR "bronchial microbiome" OR "bronchial microbiota" OR "bronchial microorganism" OR "bronchial microbe" OR "bronchial germ" OR "bronchial microbiology" OR "bronchic microbiome" OR "bronchic microbiota" OR "bronchic microorganism" OR "bronchic microbe" OR "bronchic germ" OR "bronchic microbiology" ) ) AND PUBYEAR > 2000 AND PUBYEAR < 2026 AND ( LIMIT-TO ( DOCTYPE , "ar" ) OR LIMIT-TO ( DOCTYPE , "re" ) ) AND ( LIMIT-TO ( LANGUAGE , "English" ) ).
